# Application of machine learning techniques for predicting survival in ovarian cancer

**DOI:** 10.1186/s12911-022-02087-y

**Published:** 2022-12-30

**Authors:** Amir Sorayaie Azar, Samin Babaei Rikan, Amin Naemi, Jamshid Bagherzadeh Mohasefi, Habibollah Pirnejad, Matin Bagherzadeh Mohasefi, Uffe Kock Wiil

**Affiliations:** 1 Department of Computer Engineering, Urmia University, Urmia, Iran; 2 Center for Health Informatics and Technology, The Maersk Mc-Kinney Moller Institute, University of Southern Denmark, Odense, Denmark; 3 Patient Safety Research Center, Clinical Research Institute, Urmia University of Medical Sciences, Urmia, Iran; 4 Erasmus School of Health Policy and Management (ESHPM), Erasmus University Rotterdam, Rotterdam, The Netherlands; 5 School of Medicine, University of Bari-Aldo Moro, Bari, Italy

**Keywords:** Ovarian cancer, Clinical features, Survival prediction, Machine learning, Interpretable machine learning

## Abstract

**Background:**

Ovarian cancer is the fifth leading cause of mortality among women in the United States. Ovarian cancer is also known as forgotten cancer or silent disease. The survival of ovarian cancer patients depends on several factors, including the treatment process and the prognosis.

**Methods:**

The ovarian cancer patients’ dataset is compiled from the Surveillance, Epidemiology, and End Results (SEER) database. With the help of a clinician, the dataset is curated, and the most relevant features are selected. Pearson’s second coefficient of skewness test is used to evaluate the skewness of the dataset. Pearson correlation coefficient is also used to investigate the associations between features. Statistical test is utilized to evaluate the significance of the features. Six Machine Learning (ML) models, including K-Nearest Neighbors , Support Vector Machine (SVM), Decision Tree (DT), Random Forest (RF), Adaptive Boosting (AdaBoost), and Extreme Gradient Boosting (XGBoost), are implemented for survival prediction in both classification and regression approaches. An interpretable method, Shapley Additive Explanations (SHAP), is applied to clarify the decision-making process and determine the importance of each feature in prediction. Additionally, DTs of the RF model are displayed to show how the model predicts the survival intervals.

**Results:**

Our results show that RF (Accuracy = 88.72%, AUC = 82.38%) and XGBoost (Root Mean Squad Error (RMSE)) = 20.61%, *R*^2^ = 0.4667) have the best performance for classification and regression approaches, respectively. Furthermore, using the SHAP method along with extracted DTs of the RF model, the most important features in the dataset are identified. Histologic type ICD-O-3, chemotherapy recode, year of diagnosis, age at diagnosis, tumor stage, and grade are the most important determinant factors in survival prediction.

**Conclusion:**

To the best of our knowledge, our study is the first study that develops various ML models to predict ovarian cancer patients’ survival on the SEER database in both classification and regression approaches. These ML algorithms also achieve more accurate results and outperform statistical methods. Furthermore, our study is the first study to use the SHAP method to increase confidence and transparency of the proposed models’ prediction for clinicians. Moreover, our developed models, as an automated auxiliary tool, can help clinicians to have a better understanding of the estimated survival as well as important features that affect survival.

## Background

Cancer is the second deadliest disease around the world [1]. Due to the Coronavirus Disease 2019 (COVID-19) pandemic [2], cancer diagnosis and treatment were hindered [3]. With transforming and reorganizing healthcare systems to overcome COVID-19 difficulties, the screening, diagnosis, and treatment of cancers were not considered sufficiently. As a result, the pandemic raised serious concerns about the progression and increased mortality of cancers because clinicians do not have a tool for prioritizing high-risk patients in such a low-resource condition [4].

Ovarian cancer has a poor prognosis in most women since it is diagnosed at advanced stages [5]. This cancer is called forgotten cancer and is sometimes misdiagnosed [5]. Ovarian cancer has the fifth highest mortality rate among women living in the United States (US) [6]. The incidence rate of this cancer was 10.9 per 100,000 women in 2014–2018, and its mortality rate was 6.5 per 100,000 women in 2015–2019 [7]. It was estimated that new cases of ovarian cancer would be 21,410, accounting for 1.1% of all new cancer cases, and the estimated deaths due to ovarian cancer would be 13,770, accounting for 2.3% of all cancer deaths in the US in 2021 [4, 7].

Prognosis and survival prediction estimate the likelihood of recovery from a disease based on a patient’s clinical condition [8]. Determining a disease prognosis plays an important role, especially in malignant diseases such as cancer. It is one of the most important elements that help clinicians decide on more appropriate treatments. Survival prediction helps patients be informed about treatment decisions and reduce their anxiety [9].

Different methods have been used to predict cancer prognosis [10–15]. Some of these studies have paid attention to ovarian cancer, and researchers have utilized statistical methods to predict the survival of patients with ovarian cancer [11–13]. For instance, Stenzel et al. [12] analyzed the overall survival of ovarian cancer patients using a multivariable Cox proportional hazard model on the Surveillance, Epidemiology, and End Results (SEER) dataset. The results showed a 28% increased mortality risk in non-Hispanic black women compared to non-Hispanic white women. They also observed no difference in the risk of mortality between the survival of Hispanic women and non-Hispanic white women. Rutten et al. [13] predicted the five-year survival of ovarian cancer using a Cox proportional hazard model. Dataset was collected from three registries that included ovarian cancer patients who received bulking surgery. They also developed a nomogram to predict one-year, three-year, and five-year survival of ovarian cancer patients. The c-statistic their model achieved was 0.71. However, in statistical methods, all samples in datasets are not utilized, and the wrong sampling method could lead to data misinterpretation. Moreover, the statistical models are not explainable.

In addition to statistical techniques, the use of Machine Learning (ML) algorithms in the field of healthcare and medicine, to solve problems with different procedure and perspective, are growing dramatically [16]. Applying ML algorithms for predicting the survival of cancer patients is a relatively new field of study. Almost ML models are explainable models that use all samples of the training dataset and their output could be non-binary. Furthermore, they can be used for both classification and regression approaches simultaneously. Black box ML models are models that have low interpretability and transparency and the clinicians cannot see the models’ decision-making process. To address these challenges interpretable ML techniques have been developed and used to explain the process of predictions for black box ML models. Therefore, ML models can be made more understandable and reliable by using interpretable ML methods [17]. Shapley Additive Explanations (SHAP) is one of the methods based on game theory to interpret and explain the ML black box models. These models are used in various applications, including diagnosing, treating, and prognosis of different types of cancers. The literature shows that utilizing ML techniques in this domain has been promising, and there is some evidence that ML algorithms can outperform traditional statistical models [18–24].

In recent years, ovarian cancer has attracted researchers’ attention, and some studies developed ML classifiers to predict patients’ survival [14, 15]. For instance, Chen [14] used L2-regularized logistic regression to predict the mortality rate of fewer than 20 months of ovarian cancer patients in the SEER dataset. They achieved 0.62 for the Area Under the Curve (AUC) metric. Grimley et al. [15] used two datasets of ovarian carcinomas from the SEER database. The first dataset contained cases that had been staged for the extent of the tumor using T, N, and M criteria, and the second dataset was a derivative of the first one by treating age, histologic type, and grade as additional factors. They generated prognostic groups with the depiction in dendrograms using the Ensemble Algorithm for Clustering Cancer Data. Results revealed that the C-index of the International Federation of Gynecology and Obstetrics staging system was 0.7371, which is slightly lower than the C-index of 0.7391 from the Ensemble Algorithm for Clustering Cancer Data in the first dataset. The analysis of the second dataset revealed that the A and H could be smoothly integrated with the T, N, and M criteria. Survival data were classified into nine prognostic groups with a C-index of 0.7605. Nevertheless, our literature review showed that there are some research aspects that need further research. The identified research gaps in this field are as follows:

- SEER database features have not been studied clinically to extract the most relevant features affecting the survival. In health informatics and bioinformatics fields of research, it is important that the parts of the study, including the dataset and its features, are clinically reliable.
- In most studies, the dataset size is small and the number of samples used is low.
- Even though the existing dataset is imbalanced, balancing techniques that can have a constructive impact on the performance of ML models have not been considered.
- Statistical techniques, which are used to explain the relationship between variables, have been the main technique; and accurate prediction for survival has not been performed.
- Classification is the common approach used for survival prediction, and there is no study utilizing a regression approach for predicting ovarian cancer patients’ survival.
- Only binary classification was used; and multiple clinically meaningful classes have not been considered for different survival time intervals of ovarian cancer patients.
- Interpretability and explainability of ML models in this field of research have not been addressed in previous studies.

In this study, six ML models of K-Nearest Neighbors (KNN), Support Vector Machine (SVM), Decision Tree (DT), Random Forest (RF), Adaptive Boosting (AdaBoost), and Extreme Gradient Boosting (XGBoost) were developed to predict the survival of ovarian cancer patients in two approaches of classification and regression [25–27]. KNN uses proximity to make predictions. It votes for the most frequent label among the nearest neighbors in classification and the average of the labels of the nearest neighbors in regression [26]. SVM uses a hyperplane to classify the data points. This algorithm aims to maximize the margin of dissociation between classes [26]. DT is an ML algorithm with a tree structure that consists of branches and nodes that illustrates every possible solution for a problem. This algorithm uses impurity metrics to make decisions [26, 27]. RF algorithm is an ensemble of multiple DTs, and the final outcome of RF is the aggregation of the DTs’ results [26]. AdaBoost is an ensemble of multiple classifiers or regressors. The final outcome of this algorithm is the combination of the results of its classifiers or regressors [26]. XGBoost consists of multiple weaker classifiers or regressors, the results of which are combined to determine the final output [25, 27]. It is the first time these algorithms are used to predict the survival of ovarian cancer patients based on the SEER database with classification and regression approaches simultaneously. These algorithms were modified since the best combination of hyperparameters was found using Grid Search. Grid Search is a method that evaluates all possible combinations of hyperparameters and picks the combination with the best results.

### Objectives and contributions

The current study is designed to address the mentioned research gaps in this domain. Therefore, it aims to provide models in both classification and regression approaches to determine the survival time period classes and the number of survival months using ML algorithms. The proposed methods consist of a modified RF algorithm for the classification approach and a modified XGBoost algorithm for the regression approach. In summary, the followings are the main contributions of this study:

- Feature engineering for SEER dataset is done based on discussion between engineers and clinicians of this study.
- It is the first study that predicts ovarian cancer survival using both classification and regression approaches.
- It is the first study that utilizes the mentioned six ML models to predict ovarian cancer patients’ survival based on the SEER dataset.
- Classification and regression models of ML are used to accurately predict the number of survival months and the survival class of the patients, respectively.
- Classes are defined based on clinical guidelines to have meaningful results from a clinical point of view.
- Imbalanced data issue is addressed for the classification approach to enhance the performance of the models.
- Models tuning are applied using Grid Search to find the best hyperparameters for the proposed ML models.
- An ML interpretable method has been used to explain how models make decisions to increase the chance of deploying these models in real practice.

## Materials and methods

**Fig. 1:**
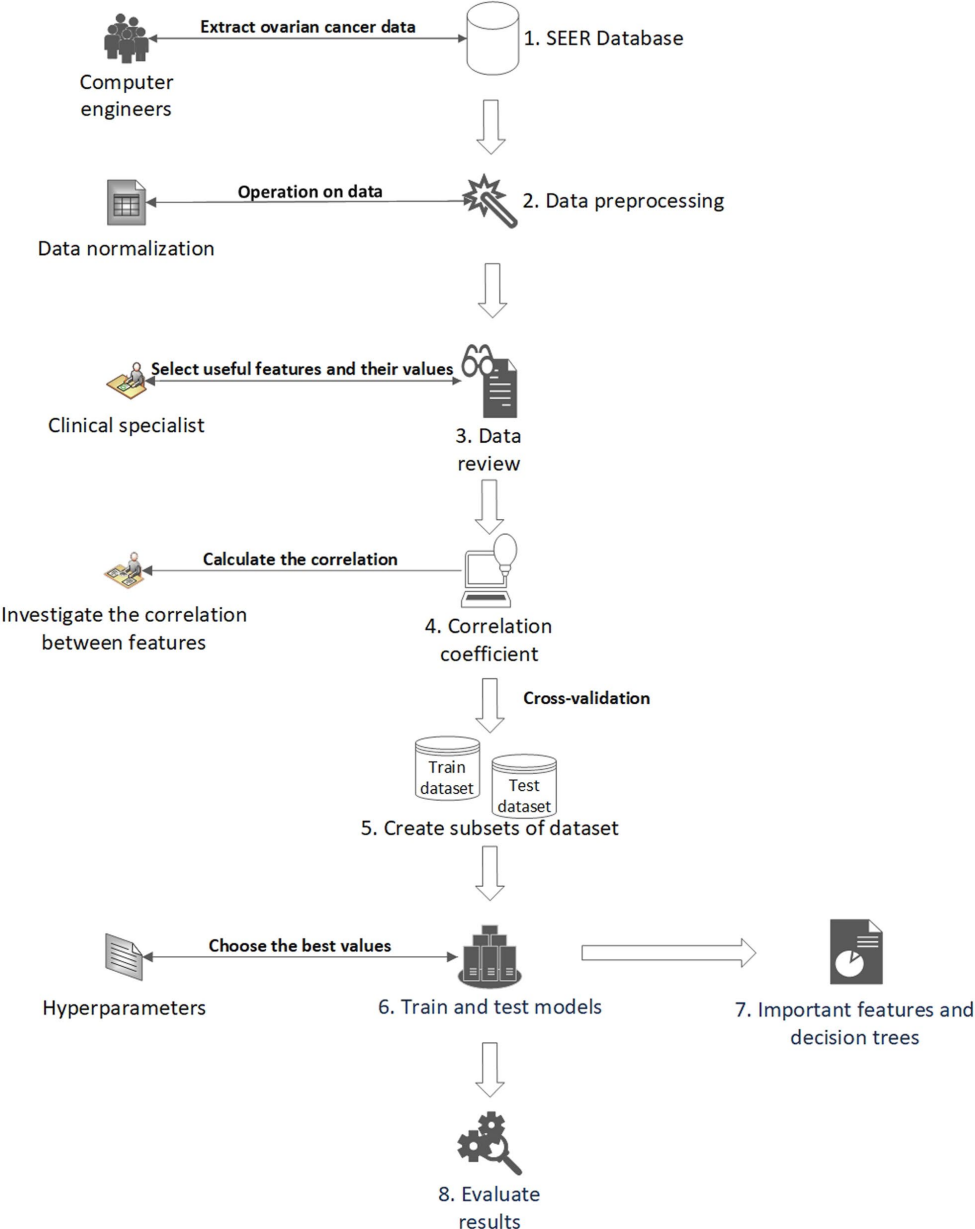
Diagram of the steps performed in this study

The methodology of this study is illustrated in Fig. 1. Different components of our method are described in the following sections.

### Study design and cohort selection

There are serious concerns about reporting the findings of utilizing ML in the health domain, and there are ongoing debates that most of the studies are not reproducible, and very difficult to judge their methodology and results. To address this issue, we followed transparent reporting of a multivariable prediction model for individual prognosis or diagnosis (TRIPOD) checklist [28, 29]. In this study, ovarian cancer data were collected from the SEER database. The SEER Stat program provides a database of cancer-related details and statistics. The SEER database is one of the most comprehensive and extensive population-based cancer registry data [4]. Ovarian cancer patients’ data between 2000 and 2016 were used in this study.

### Outcome

For the classification approach, five clinically meaningful classes of survival namely, Class 0 (zero to 6 months), Class 1 (6 months to one year), Class 2 (one to three years), Class 3 (three to five years), Class 4 (five years and older) were defined. For the regression approach, the survival time in the number of months was considered.

### Data preparation

SEER database is one of the most comprehensive and largest cancer databases that contains information on cancer patients of 48% of the US population [30]. Various information like data on tumor morphology and stage at diagnosis, primary tumor site, patient demographics, the first course of treatment, and follow-up for vital status (survival) from the 22 geographic areas of the US has been collected. It is supported by the Surveillance Research Program in National Cancer Institution’s Division of Cancer Control and Population Sciences [31].

For this study, the data of ovarian cancer patients between 2000 and 2016 was picked from the SEER database. This database contained many features; therefore, we performed feature selection. Feature selection is the process of removing irrelevant features and noise from the dataset to increase the accuracy and predictive power and decrease the learning time of the models. We performed feature selection under the supervision of a clinical researcher. So clinically significant and necessary features in predicting the survival of ovarian cancer patients were selected. The final dataset contained 42,827 samples and 17 features, as shown in Table 1. The survival months in this dataset were between 0 and 198 months.

**Table 1:** Dataset description

Feature	Domain of values	Type of feature
County	165 different values	Categorical
Histologic type ICD-O-3	150 different values	Categorical
Laterality	1. Bilateral, single primary 2. Paired site, but no information concerning laterality 3. Right - origin of primary 4. Left - origin of primary 5. Only one side - side unspecified	Categorical
Radiation sequence with surgery	1. No radiation and/or cancer-directed surgery 2. Radiation after surgery 3. Radiation prior to surgery 4. Sequence unknown, but both were given 5. Radiation before and after surgery 6. Intraoperative radiation	Categorical
Reason no cancer-directed surgery	1. Surgery performed 2. Not recommended 3. Recommended but not performed, unknown reason 4. Not recommended, contraindicated due to other cond; autopsy only 5. Not performed, patient died prior to recommended surgery	Categorical
Sequence number	1. One primary only 2. 1st of 2 or more primaries	Categorical
Race recode	1. White 2. Black 3. Asian or Pacific Islander 4. American Indian/Alaska Native	Categorical
Marital status at diagnosis	1. Married (including common law) 2. Widowed 3. Single (never married) 4. Divorced 5. Separated 6. Unmarried or Domestic Partner	Categorical
PRCDA region	1. Pacific Coast 2. East 3. Northern Plains 4. Southwest 5. Alaska	Categorical
Summary stage	1. Distant 2. Regional 3. Localized	Categorical
Insurance recode	1. Insured 2. Insured/No specifics 3. Any Medicaid 4. Insurance status unknown 5. Uninsured	Categorical
CS site-specific factor 1	6 different numeric values (Mean:509, Standard deviation: 535.71, Range: 10–999)	Numerical
Year of diagnosis	17 different years (Range: 2000–2016)	Numerical
Age at diagnosis	100 different ages (Range: 0-113)	Numerical
Chemotherapy recode	1. yes 2. no	Categorical
Rural-Urban continuum code	1. Counties in metropolitan areas GE 1 million pop 2. Counties in metropolitan areas of 250,000 to 1 million pop 3. Counties in metropolitan areas of LT 250 thousand pop 4. Urban pop of 2,500 to 19,999, adjacent to a metro area 5. Urban pop of 2,500 to 19,999, not adjacent to a metro area 6. Urban pop of GE 20,000 adjacent to a metropolitan area 7. Urban pop of GE 20,000 not adjacent to a metropolitan area 8. Comp rural LT 2,500 urban pop, not adjacent to metro area 9. Comp rural LT 2,500 urban pop, adjacent to a metro area	Categorical
Grade	1. Well differentiated; Grade I 2. Moderately differentiated; Grade II 3. Poorly differentiated; Grade III 4. Undifferentiated; anaplastic; Grade IV	Categorical

Then, we used the Min-Max normalization method from the Sklearn library of Python. Doing so, the data was mapped to the range of (0,1) according to Eq. (1), where ${X}_{min}$ is the minimum value, and ${X}_{max}$ is the maximum value of a feature in the dataset.

1 $${X}_{normalize}=\frac{X-{X}_{min}}{{X}_{max}-{X}_{min}}$$

The survival of ovarian cancer has been evaluated in terms of 1, 3, 5, and 10 years following the diagnosis of cancer [32, 33]. However, shorter intervals can be selected to make survival predictions more precise. Therefore, for the classification approach, five classes of survival months, namely Class 0 (zero to six months), Class 1 (six months to one year), Class 2 (one year to three years), Class 3 (three years to five years), and Class 4 (five years and above) were considered. Furthermore, to examine the survival of ovarian cancer patients accurately, patients’ survival in the number of months was also predicted using the regression approach.

### Feature importance

The collected dataset included many numerical and categorical features. Based on discussions with clinicians, irrelevant features and incomplete records were removed from the dataset. Then, pearson correlation coefficient was used to investigate the associations between all features of the dataset. The correlation coefficient determines the degree and type of pairwise associations between features [34]. These coefficients have values between − 1 and 1, where 1 is the maximum correlation.

Determining the features’ importance of a dataset can be useful to support medical decisions and improve the patients’ quality of treatment [35]. It can be effective in predicting the survival of cancer patients to help clinicians make decisions by visualizing how decisions are made in the models [36, 37]. Therefore, we applied the SHAP library to interpret the model on our dataset. This vision and insight determine each feature’s importance and effectiveness in decision-making within the model [38].

### Data imbalance

Data skewness measures the asymmetry of the distribution of a dataset. It is one of the inevitable challenges in many datasets, especially medical datasets. Skewed data can lead to a non-uniform sampling of the target feature and have an adverse effect on the performance of ML models. To solve this issue, Synthetic Minority Oversampling Technique (SMOTE) was used. SMOTE is a technique that creates synthetic data to oversample the minority classes in a dataset [39, 40]. After balancing the dataset, we have 14,778 samples for each class resulting in a total of 73,890 samples for all classes in the classification approach. One of the methods of testing the skewness of dataset is Pearson’s second coefficient of skewness test. This method determines the symmetry of the distribution of the dataset. Therefore, according to Eq. (2), this skewness test method was used to show the symmetry of the distribution of our dataset [41].

2 $$Skewness=\frac{3\times \left(\overline{x}-m\right)}{s}$$

In Eq. (2), $\overline{x}$ is the mean, $m$ is the median, and $s$ is the standard deviation.

### Predictive models development

The KNN algorithm is one of the simplest supervised ML algorithms. This algorithm processes all the samples of training to make a prediction. For the classification approach, it finds the K-nearest neighbors and predicts the class with the majority of votes of the nearest neighbors. For the regression approach, this algorithm finds the K-nearest neighbors and predicts the desired value by calculating the average value of the nearest neighbors [26].

The SVM algorithm is an instance-based and supervised ML method that separates data samples using hyperplanes. It maximizes the margin between classes. The samples on one side of the line are considered similar and have the same category. This algorithm is used for both classification and regression approaches [26]. Equation (3) shows SVM’s optimization problem.

3 $$C\left\| W \right\|^{2} + \frac{1}{N}\sum _{{i = 1}}^{N} max\left( {{\text{0,1}} - y_{i} \left( {WX_{i} - b} \right)} \right)$$

In Eq. (3), the tradeoff between guaranteeing that samples are located on the correct side of decision boundaries and expanding the size of decision boundaries is $C$, and the number of samples is $N$. To create non-linear boundaries, SVM uses kernels. The most common kernel is the Radial Basis Function kernel which is shown in Eq. (4).

4 $$k\left( {X,\mathop X\limits^{\prime } } \right) = exp\left( {\frac{{ - \left\| {X - \mathop X\limits^{\prime } } \right\|^{2} }}{{2\sigma ^{2} }}} \right)$$

In Eq. (4), the Euclidean distance of two vectors is $\left\| {X - \mathop X\limits^{\prime } } \right\|^{2}$.

The DT algorithm is a widely used supervised ML algorithm that can be used in both classification and regression approaches. This algorithm determines which strategy is most likely to be successful using the Gini criterion, which is shown in Eq. (5). This algorithm consists of leaves, roots, and branches [26, 27].

5 $$Gini \left(t\right)=1-\sum _{i=1}P{\left(\left.i\right|t\right)}^{2}$$

In Eq. (5) ratio of class at the node of $i$ is $P$.

The RF algorithm is one of the most popular supervised ML algorithms. This algorithm is used for classification and regression approaches. To reach a more robust performance, it considers many trees instead of relying on one DT and makes predictions from each tree based on the majority vote. More trees in this algorithm often improve performance and prevent over-fitting. This algorithm is one of the most effective algorithms of ML in many applications [26].

The AdaBoost algorithm is a supervised ML technique that belongs to the boosting family of algorithms. In this algorithm, a problem is predicted by several different classifiers or regressors, usually DT, and the results are combined to determine the final result for the problem. The training of the classifiers or regressors of this algorithm is sequential, which means they are trained based on the result of the previous classifier or regressor [26].

One of the most powerful supervised ML algorithms is the XGBoost algorithm. This algorithm is an enhanced form of Gradient Boosting (GB). In this algorithm, several different classifiers or regressors predict a problem, and the combination of the results is the final result for the problem. Compared with GB, the model generalization capabilities of XGBoost are better due to the advanced regularization. It is also faster and more efficient than GB. This algorithm is used to predict classification and regression approaches [25, 27].

Moreover, five-fold cross-validation was applied to prevent over-fitting and evaluate the performance of implemented models [42]. In each iteration, 80% of the dataset was dedicated for training, and the rest part was used for testing.

This study determined a range of hyperparameters’ values for experimental implementation. They are shown in Table 10 in Appendix 1. Using the Grid Search method, the optimal hyperparameters of all models were achieved. The best hyperparameters selected by this method are shown in Tables 2 and 3 for the classification and regression approaches, respectively.

**Table 2:** The best hyperparameters of models for the classification approach

Model	Hyperparameters
KNN	Algorithm: kd_tree, p: 2, n_neighbors: 2
SVM	Kernel: rbf, gamma: 0.1, C: 1
DT	Spliter: best, max_depth: none, criterion: gini
RF	Criterion: gini, max_depth: none, n_estimators: 100
AdaBoost	n_estimators: 100, learning_rate: 1.5, algorithm: SAMME.R
XGBoost	Sampling method: gradiant_based, eta: 0.5, booster: gbtree

**Table 3:** The best hyperparameters of models for the regression approach

Model	Hyperparameters
KNN	Algorithm: ball_tree, p: 2, n_neighbors: 14
SVM	Kernel: rbf, gamma: 1, C: 10
DT	Spliter: best, max_depth: none, criterion: gini
RF	Criterion: gini, max_depth: none, n_estimators: 150
AdaBoost	n_estimators: 50, learning_rate: 0.5, algorithm: SAMME
XGBoost	Sampling method: uniform, eta: 0.3, booster: gbtree

### Predictive models evaluation

Various performance metrics were used for the evaluation of ML models. Ordinary Least Squares is a type of linear least-squares method to estimate the parameters in a linear regression model that describes the relationship between independent quantitative features and dependent features [43, 44]. It is used to show the statistically significant difference between the values of features. This test considers statistical significance (p-value) at different levels of 0.05, 0.01, and 0.001.

The confusion matrix is one of the classification evaluation criteria. This matrix is a square matrix whose dimension is $n\times n$. The parameter n is equal to the number of classes in the classification [45]. After training and testing the models, we used the following ML evaluation criteria for the classification approach [46–48]. Accuracy is the ratio between the correct predictions of the data points to the total number of predictions. This criterion is used to evaluate the ML classification models [47]. The accuracy formula is shown in Eq. (6).

6 $$Accuracy=\frac{TP+TN}{TP+FP+FN+TN}$$

Precision or Positive Predictive Value (PPV) is one of the evaluation criteria of ML models. This criterion is the ratio between the total number of true positives to the total number of false positives and true positives. Precision indicates the accuracy of a model in a positive prediction [48]. Equation (7) shows the formula of Precision.

7 $$Precision \vee Positive\;Predictive\;Value\;\left( {PPV} \right) = \frac{{TP}}{{FP + TP}}$$

Negative predictive value (NPV) is calculated as the ratio between the total number of true negatives to the total number of false negatives and true negatives. It is one of the evaluation criteria of ML classification models that shows the probability that a person whose disease test is negative is truly healthy [48]. NPV formula is shown in Eq. (8).

8 $$Negative\;Predictive\;Value\;\left( {NPV} \right) = \frac{{TN}}{{FN + TN}}$$

Sensitivity or Recall is the ratio between the total number of true positives to the total number of false negatives and true positives. This criterion is used to evaluate ML models and the model’s ability in detecting positive samples [46]. The sensitivity formula is shown in Eq. (9).

9 $$Sensitivity\vee Recall=\frac{TP}{FN+TP}$$

Specificity is defined as the ratio between the total number of true negatives to the total number of false positives and true negatives. It is used in the performance evaluation of ML models. This criterion is important when the negative cases have priority. Because it shows the ability of the model to correctly detect true negatives. Equation (10) shows the formula of Specificity [46].

10 $$Specificity=\frac{TN}{FP+TN}$$

F1-Score calculates the harmonic mean of recall and precision and combines them into one metric so that models can be compared with one metric. A high F1-Score indicates low false positives and low false negatives [46, 48]. The F1-Score formula is shown in Eq. (11).

11 $$F1-Score=\frac{2\times Precision\times Recall}{Precision+Recall}$$

In Eqs. (6–10), TP stands for true positive, TN stands for true negative, FP stands for false positive, and FN stands for false negative.

The AUC means the area bounded by the Receiver Operating Characteristic curve. It is a measure of the overall accuracy of the models. The results of AUC range from 0 to 1, where a low value means bad model performance and a high value means an accurate model [47].

We also used the following evaluation criteria for the regression approach (prediction and prognosis of patients’ survival months). Root Mean Square Error (RMSE) calculates the standard deviation of the prediction errors. Prediction errors are the distance between the regression line and the data points. It measures the data’s concentration around the regression line. RMSE’s value is always non-negative, and a lower RMSE value is better than a higher value [49]. Equation (12) shows the formula of RMSE.

12 $$Root Mean Square Error \left(RMSE\right)=\sqrt{\frac{1}{m}\times {\sum }_{i=1}^{m}{\left({X}_{i}-{Y}_{i}\right)}^{2}}$$

In Eq. (12), $i$ is a sample, $m$ means the number of samples, ${X}_{i}$ shows the actual target value for sample $i$, and ${Y}_{i}$ denotes the predicted target value for sample $i$.

R-Squared (R^2^) is a statistical measure that is used in the regression. It determines the proportion of variance in the dependent variable that is explained by the independent variable. R² indicates how well the regression model fits the data. Higher R² indicates that the relationship between the dependent variables and the regression model is strong [50]. The R² formula is shown in Eq. (13).

13 $$R-Squared \left({R}^{2}\right)=\frac{TSS-RSS}{TSS}$$

In Eq. (13), $TSS$means the total sum of squares and $RSS$means the residual sum of squares.

## Results

Using the Pearson correlation coefficient, the correlation between features of the primary dataset was investigated. Some of these features had correlation coefficient values greater than 0.5 and consequently had a strong association with each other. The year of diagnosis was related to the insurance record with a correlation coefficient value of 0.85. Furthermore, the year of diagnosis was related to the CS site-specific factor 1 with a correlation coefficient value of 0.52. For other features, the correlation coefficient values were less than 0.5. Moreover, there was no complete inverse relevance between the features.

Furthermore, the skewness test was conducted in this study, where values greater than 1 and less than − 1 indicate high skewness. The initial value for the original dataset was 0.213, which is acceptable, but it can be improved by using a proper balancing technique. Therefore, SMOTE was applied to the original dataset, and the skewness was measured. The skewness value for the new dataset was − 0.032, which is closer to 0, indicating a more symmetric and balanced distribution.

Table 4 shows the comparison of the significance of the relation between the features of our dataset and the outcome variable. Some of the features were rejected at 0.05 and some at 0.01. The 0.001 level was used to ensure that there was no feature that we did not know had been rejected or accepted.

**Table 4:** Comparing the level of significance of the dataset’s features

Feature	*P-*Value < 0.05	*P-*Value < 0.01	*P-*Value < 0.001
County	Reject	Reject	Reject
Histologic type ICD-O-3	Accept	Accept	Accept
Laterality	Accept	Accept	Accept
Radiation sequence with surgery	Reject	Reject	Reject
Reason no cancer-directed surgery	Accept	Accept	Accept
Sequence number	Accept	Accept	Accept
Race recode	Accept	Accept	Accept
Marital status at diagnosis	Accept	Accept	Accept
PRCDA region	Reject	Reject	Reject
Summary stage	Accept	Accept	Accept
Insurance recode	Accept	Accept	Accept
CS site-specific factor 1	Accept	Accept	Accept
Year of diagnosis	Accept	Accept	Accept
Age at diagnosis	Accept	Accept	Accept
Chemotherapy recode	Accept	Accept	Accept
Rural-Urban continuum code	Accept	Accept	Reject
Grade	Accept	Accept	Accept

### Classification models

Accuracy, PPV, NPV, sensitivity or recall, specificity, F1-Score, and AUC were calculated to evaluate the performance of the proposed classification models. Table 5 shows the average performance of the five folds of cross-validation of the proposed models.

**Table 5:** Average performance of the five folds of the proposed classification models

Model	Class	Accuracy (%)	PPV (%)	NPV (%)	Sensitivity or recall (%)	Specificity (%)	F1-Score (%)	AUC (%)
KNN	Class 0	85.68	63.45	91.64	67.07	90.34	65.20	78.70
	Class 1	90.23	70.04	97.16	89.45	90.43	78.56	89.94
	Class 2	81.41	54.77	86.02	40.45	91.65	46.52	66.05
	Class 3	89.52	74.53	93.13	72.34	93.82	73.41	83.08
	Class 4	93.26	84.66	95.29	80.99	96.33	82.78	88.66
	*Average*	*88.02*	*69.49*	*92.65*	*70.06*	*92.51*	*69.29*	*81.29*
SVM	Class 0	83.14	56.78	91.09	65.80	87.47	60.95	76.64
	Class 1	74.69	35.30	83.36	31.85	85.39	33.48	58.62
	Class 2	78.05	37.21	81.38	13.89	94.09	20.20	53.99
	Class 3	75.78	35.80	82.74	26.51	88.11	30.45	57.31
	Class 4	70.98	37.64	90.12	68.62	71.58	48.61	70.10
	*Average*	*76.53*	*40.55*	*85.74*	*41.33*	*85.33*	*38.74*	*63.33*
DT	Class 0	83.60	59.42	89.29	56.67	90.33	58.01	73.50
	Class 1	84.38	60.52	90.67	63.08	89.71	61.77	76.39
	Class 2	76.15	39.99	84.74	38.34	85.61	39.13	61.98
	Class 3	82.09	55.18	88.91	55.80	88.67	55.48	72.23
	Class 4	86.27	65.16	91.77	67.40	90.99	66.26	79.19
	*Average*	*82.50*	*56.05*	*89.08*	*56.26*	*89.06*	*56.13*	*72.66*
RF	Class 0	88.51	69.79	93.63	74.99	91.88	72.30	83.44
	Class 1	92.09	83.16	94.08	75.81	96.16	79.31	85.98
	Class 2	81.61	54.08	88.37	53.35	88.68	53.70	71.01
	Class 3	89.21	74.04	92.81	70.95	93.78	72.46	82.37
	Class 4	92.18	78.49	95.91	83.96	94.24	81.13	89.10
	*Average*	***88.72***	***71.91***	***92.96***	***71.81***	***92.95***	***71.78***	***82.38***
AdaBoost	Class 0	85.17	63.25	90.50	61.53	91.10	62.02	76.32
	Class 1	78.13	46.17	85.55	40.85	87.46	42.77	64.16
	Class 2	75.37	38.95	84.99	40.60	84.07	39.70	62.34
	Class 3	76.70	36.97	82.46	23.43	90.02	28.67	56.72
	Class 4	75.86	42.85	89.27	61.87	79.37	50.62	70.62
	*Average*	*78.25*	*45.64*	*86.55*	*45.66*	*86.40*	*44.76*	*66.03*
XGBoost	Class 0	87.98	67.12	94.33	78.28	90.41	72.27	84.34
	Class 1	85.78	68.45	89.01	53.69	93.81	60.18	73.75
	Class 2	76.64	43.22	87.63	53.47	82.44	47.78	67.95
	Class 3	80.73	52.87	84.89	34.29	92.34	41.56	63.31
	Class 4	82.60	55.61	90.79	64.68	87.08	59.79	75.88
	*Average*	*82.75*	*57.45*	*89.33*	*56.88*	*89.22*	*56.32*	*73.05*

Similar to other studies, accuracy and AUC metrics were considered as the most important criteria for evaluating the models [51, 52]. As shown in Table 5, on average, RF had the best performance for all evaluation criteria for the classification approach, which is bolded and italicized in Table 5. Furthermore, the computing time of one cycle of each fold of classification models is shown in Table 6. The lowest computing time is bolded in Table 6.

**Table 6:** The computational time of one cycle of classification models

Model	Time (Seconds)
KNN	59.64
SVM	2109.13
DT	280.74
RF	**7.57**
AdaBoost	171.68
XGBoost	15.07

RF model achieved 88.72%, 71.91%, 92.96%, 71.81%, 92.95%, 71.78%, and 82.38%, on average, for all classes regarding the accuracy, PPV, NPV, sensitivity or recall, specificity, F1-Score, and AUC, respectively. More detailed values obtained for five folds of the RF model are illustrated in Table 11 of Appendix 1. Also, the RF model in the classification approach achieved the lowest execution time in one-fold among all other proposed models.

**Fig. 2:**
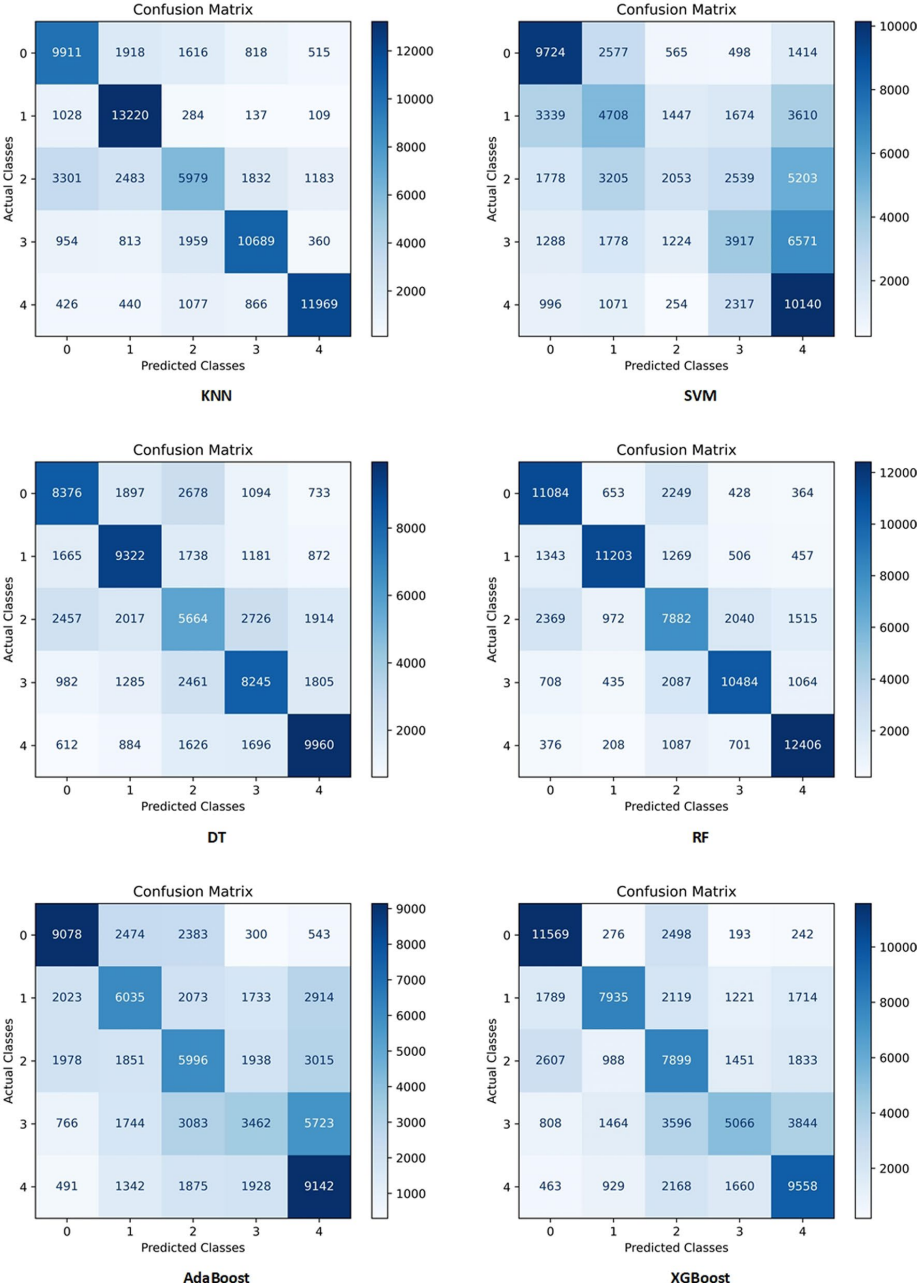
Confusion matrices of all models in the average of all folds

**Fig. 3:**
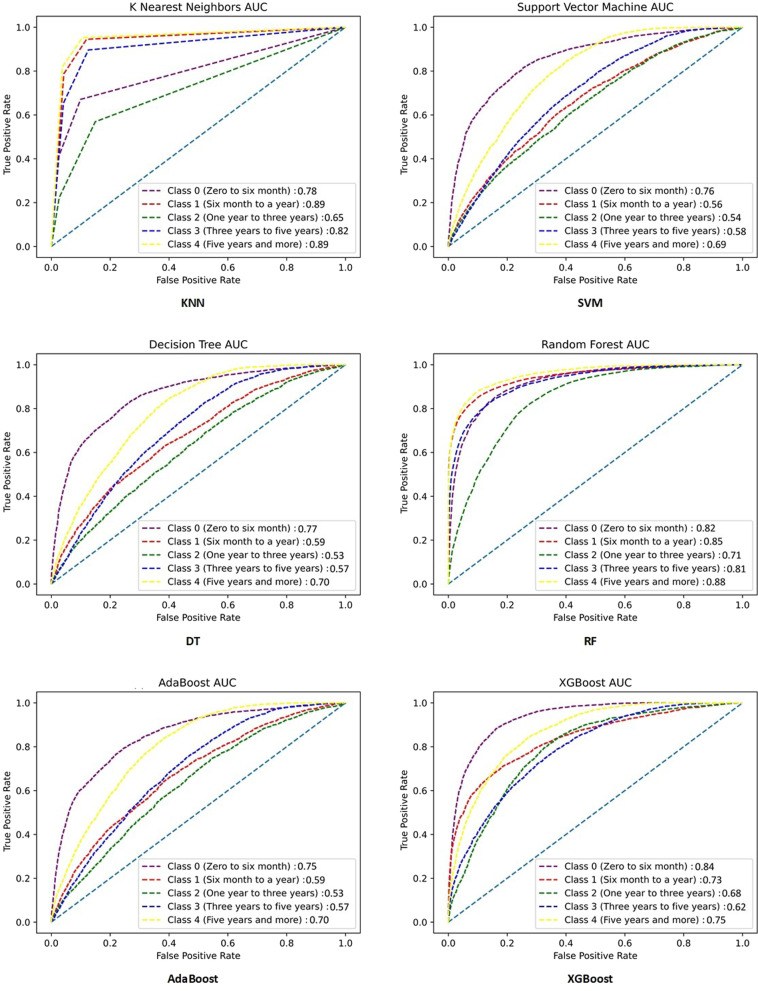
The AUC diagrams of all models in the fifth fold

Moreover, the performances of all models for all folds were compared using confusion matrices, and the results were shown in Fig. 2. The fifth fold was used for visualizing the performance of the developed models based on the AUC metric, and the results are provided in Fig. 3. Moreover, as the best model (RF model), the confusion matrices and AUC diagrams of the RF model for all folds are shown in Fig. 7 and Fig. 8 in Appendix 1, respectively.

### Regression models

To evaluate the performance of the proposed models of this study for the regression approach, the values of RMSE and R^2^ were calculated due to their importance which is in the same direction as the study’s criteria [53]. Furthermore, the computing time of one cycle of each fold of regression models is shown in Table 7. The lowest computing time is bolded in Table 7.

**Table 7:** The computational time of one cycle of regression models

Model	Time (Seconds)
KNN	15.82
SVM	102.84
DT	178.01
RF	16.26
AdaBoost	12.17
XGBoost	**0.71**

The average performance of the five folds cross-validation of the proposed models is shown in Table 8. As seen, XGBoost with RMSE = 20.61% and R^2^ = 0.4667 had the best performance for all evaluation metrics. The results of XGBoost for all five folds are listed in Table 12 in Appendix 1. Also, the XGBoost model in the regression approach achieved the lowest execution time in one-fold among all other proposed models.

**Table 8:** Average performance of the five folds of the proposed regression models

Model	RMSE (%)	R^2^
KNN	24.71	0.2372
SVM	25.62	0.1804
DT	25.14	0.2104
RF	24.25	0.2658
AdaBoost	27.76	0.3682
XGBoost	**20.61**	**0.4667**

#### Evaluation of the best-proposed models

The results showed that RF and XGBoost had the best performance for predicting the survival of ovarian cancer patients in classification and regression approaches, respectively.

**Fig. 4:**
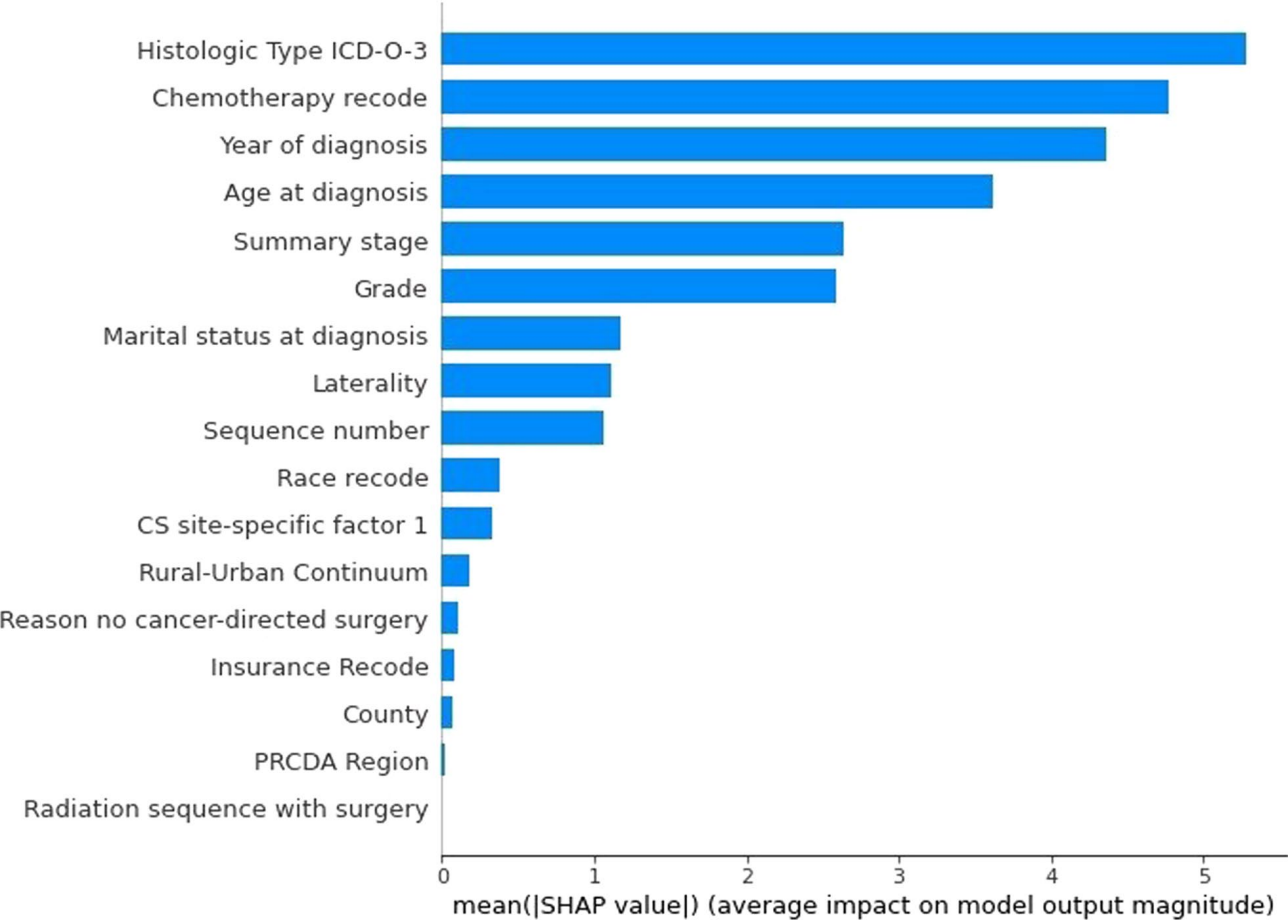
The most important features of the dataset of this study

The most important features determined by RF are shown in Fig. 4. As seen, the nine most important features with an average SHAP value greater than one are histologic type ICD-O-3, chemotherapy recode, year of diagnosis, age at diagnosis, summary stage, grade, marital status at diagnosis, laterality, and sequence number. The histologic type ICD-O-3 is the most discriminative feature for the purpose of predicting survival.

**Fig. 5:**
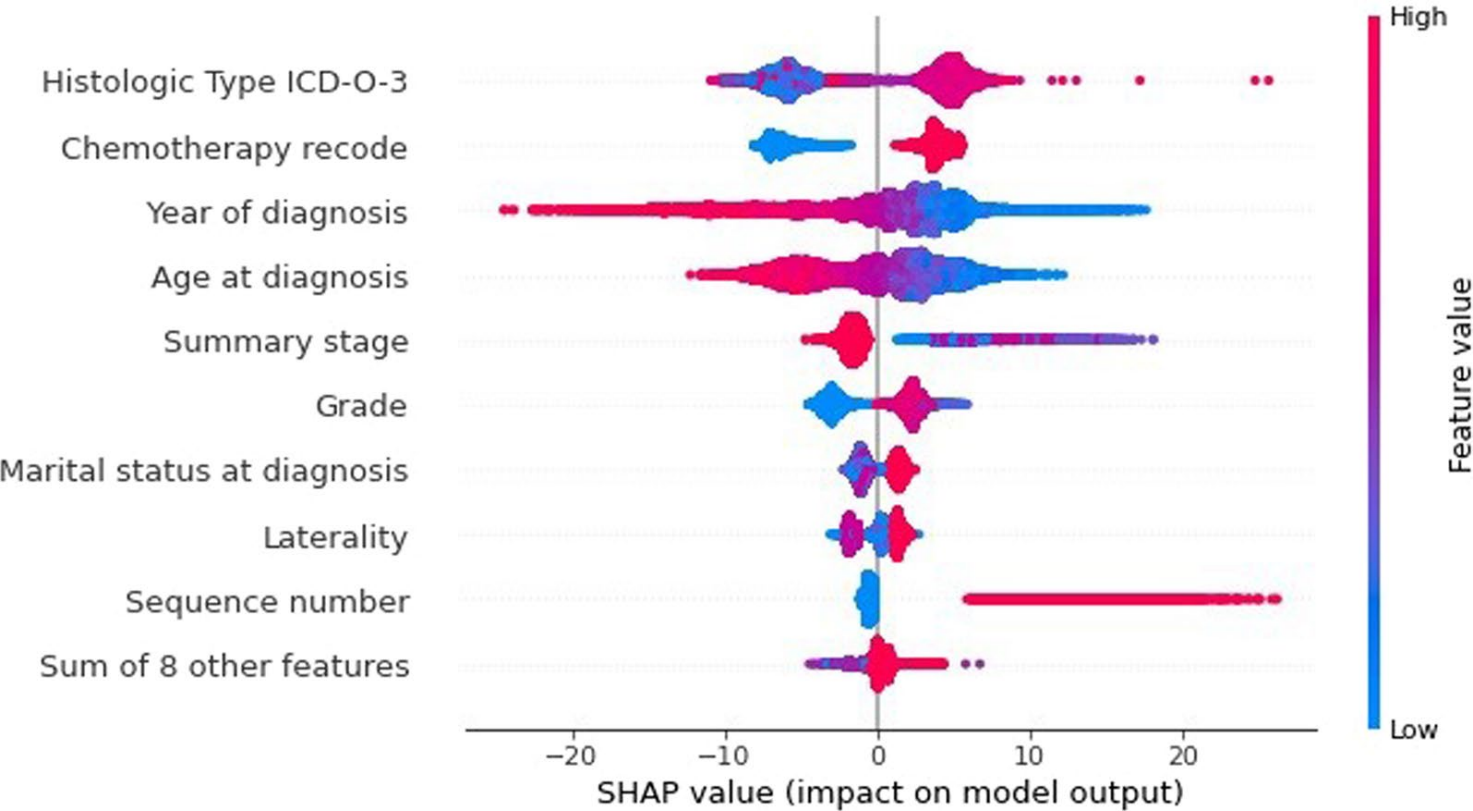
Impact of the features of the dataset on the model’s decision-making

**Fig. 6:**
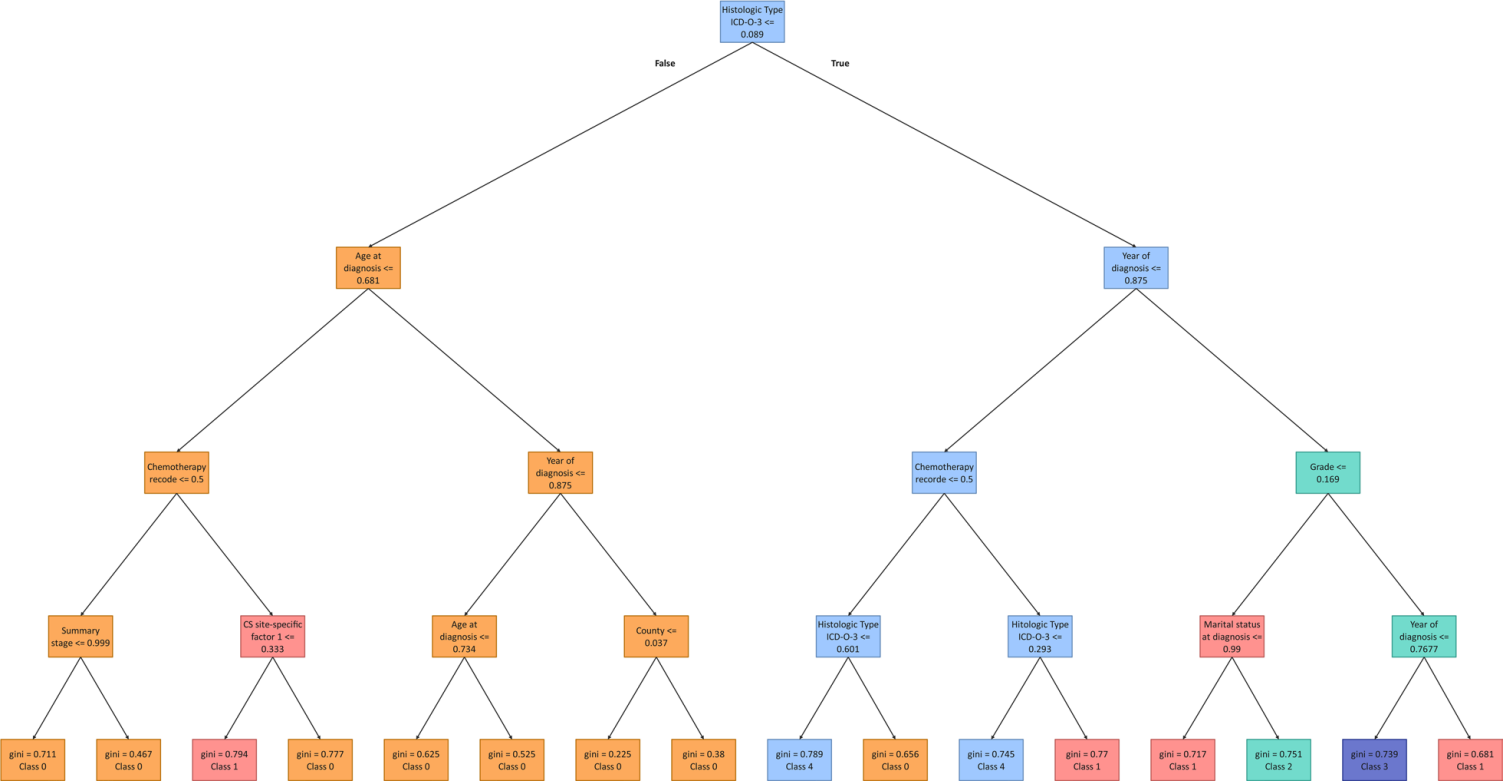
100th tree of our RF model

**Fig. 7:**
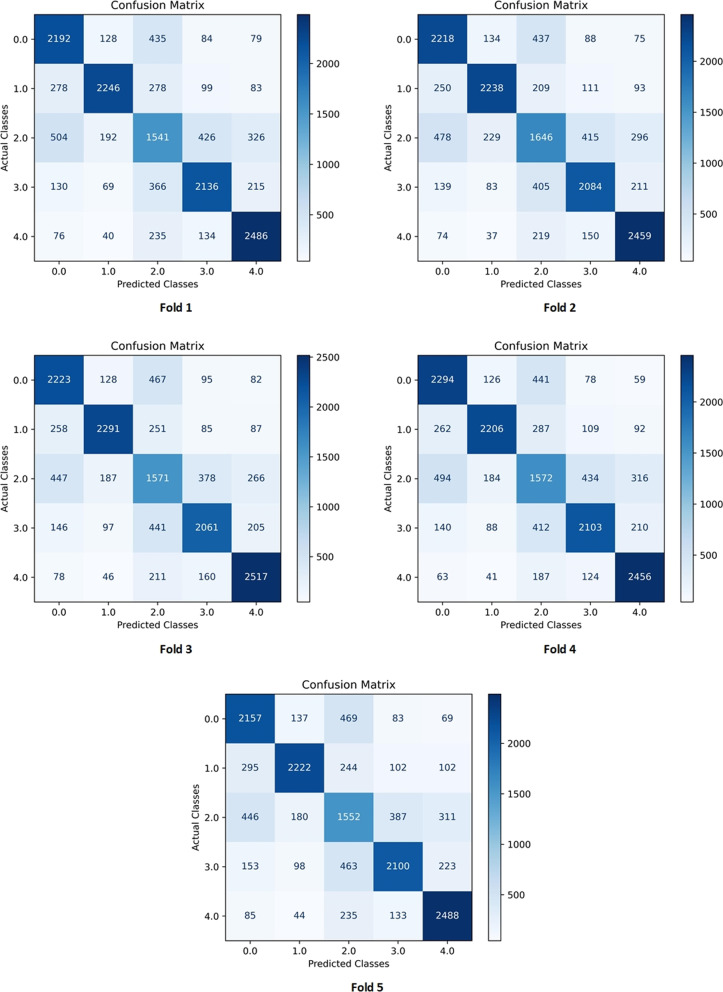
Confusion matrices of all folds of the RF model.

**Fig. 8:**
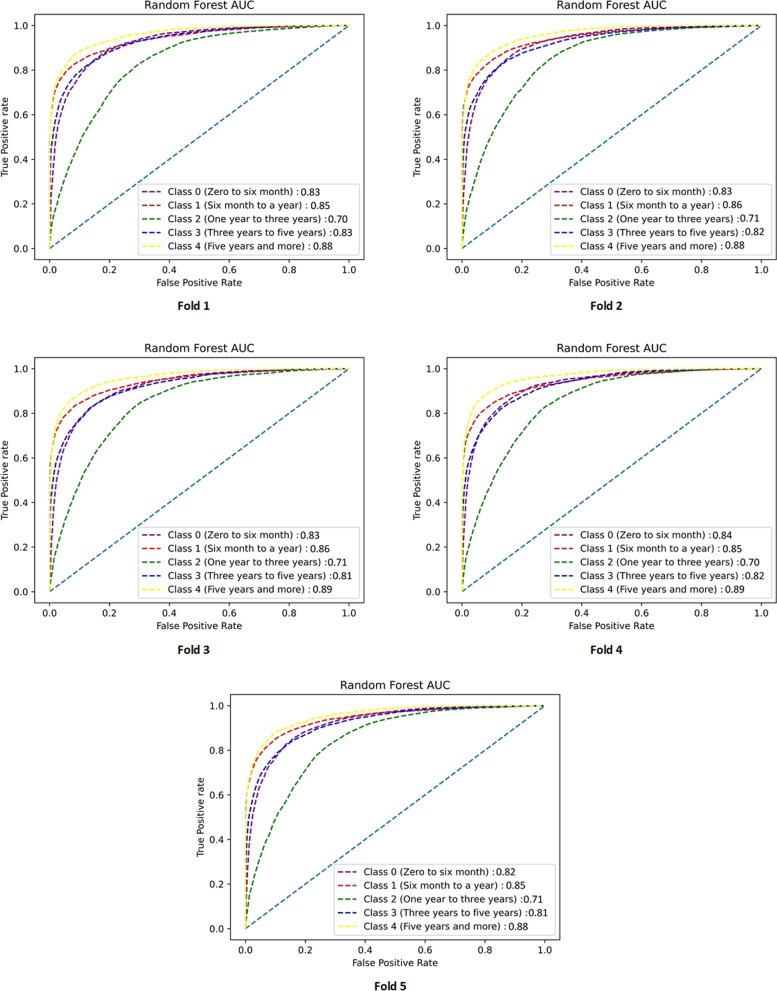
AUC of all the folds of the RF model

**Fig. 9:**
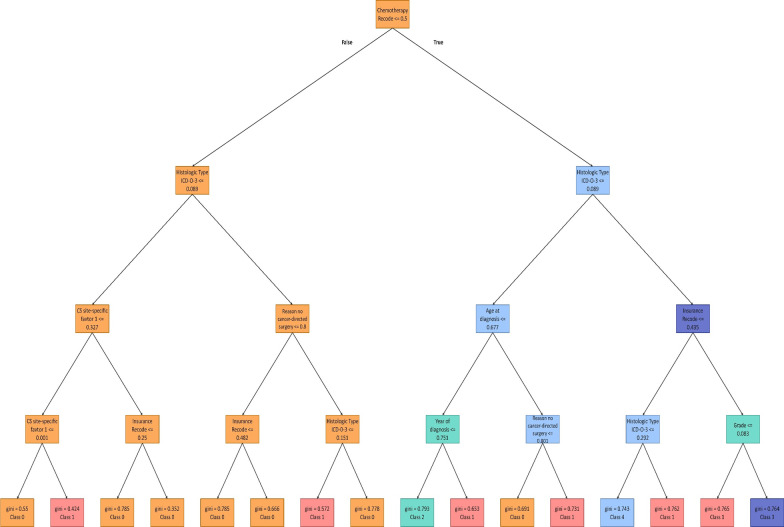
20th tree of our RF model.

**Fig. 10:**
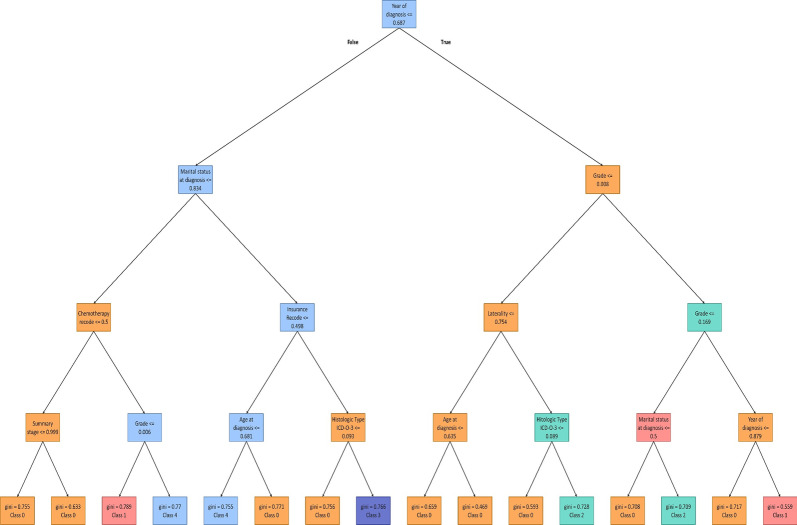
40th tree of our RF model.

**Fig. 11:**
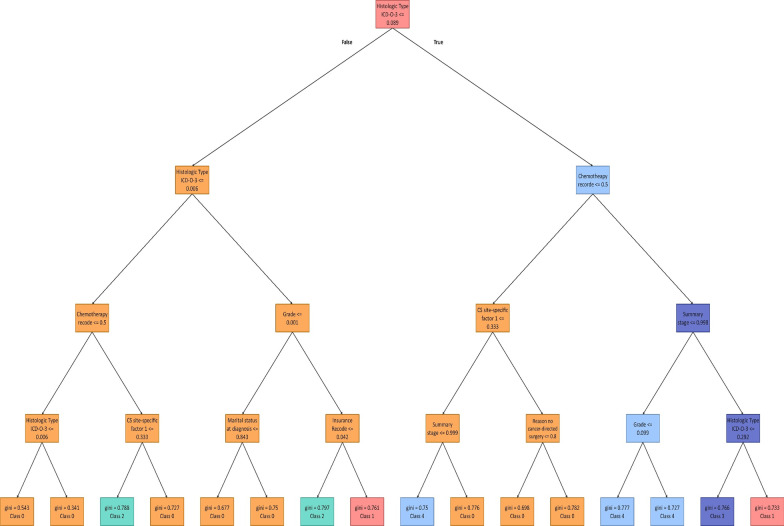
60th tree of our RF model.

**Fig. 12:**
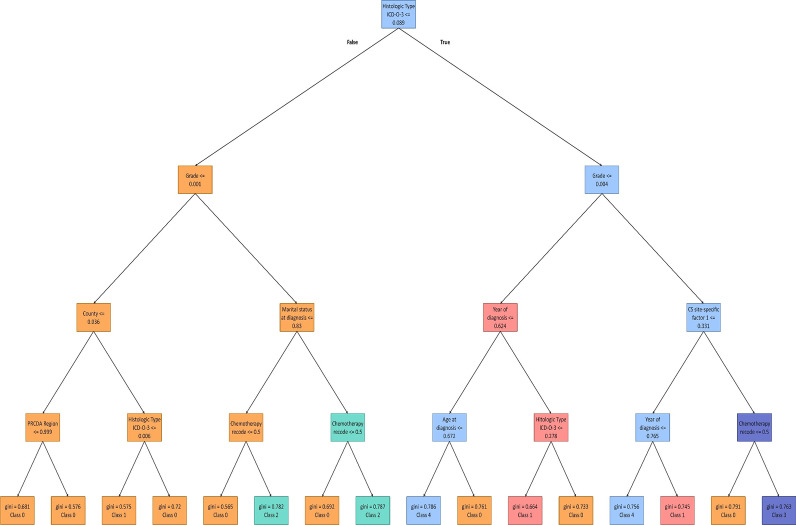
80th tree of our RF model.

Figure 5 shows the effect of nine important features of this study and their impacts on the models’ output using the SHAP value. We randomly selected the 20th, 40th, 60th, 80th, and 100th trees created by our RF model with a depth of four to display predicted classes as shown in Figs. 9, 10, 11 and 12 in Appendix 1 and Fig. 6, respectively.

## Discussion

In this study, we developed six ML models, including SVM, KNN, DT, RF, AdaBoost, and XGBoost, to predict the survival of ovarian cancer patients. For this aim, both classification and regression approaches for the SEER dataset were implemented. The promising results of this study are due to the appropriate size of the dataset’s samples and correct preprocessing. Therefore, we were able to get accurate outcomes with relatively few errors in the results [54].

The survival intervals of cancer patients are important and meaningful for clinicians, and they can plan patients’ treatment better based on that [55]. Therefore, in this study, with the help of an expert clinician, five classes including Class 0 (zero to 6 months), Class 1 (6 months to one year), Class 2 (one to three years), Class 3 (three to five years), Class 4 (five years and older) were considered. For the regression approach, the number of survival months was predicted.

Some studies have used the SEER dataset to predict ovarian cancer survival [11–15]; however, this field has some open areas that need further research. In the current study, we identified some of those gaps and addressed them. First, as interdisciplinary research, it is necessary to have a clinician in the research team to curate data, so our clinician evaluated the dataset carefully to identify the relevant features for survival prediction. Second, based on discussions with the clinician, we defined five classes that are meaningful from the clinical point of view. Third, to the best of our knowledge, the current approach in the studies is to develop classifiers; however, to have more accurate results, we implemented the regression approach to predict survival in months. Fourth, we paid attention to the interpretability and transparency of our system, so SHAP was used to explain the impact and importance of each feature on the model prediction performance. Moreover, we developed DTs of the RF technique that can help clinicians better understand the prediction process.

Our results showed that RF, with an accuracy of 88.72% and AUC of 82.38% on average, had the best performance for the classification approach, which is also better than models presented in the previous literature [14, 15]. For the regression approach, the XGBoost model with a RMSE of 20.61% and an R^2^ of 0.4667 had the best performance. Our proposed best models in each approach have the least computing time in one cycle run time compared to other proposed models.

Moreover, the performance of the ML models for each class was evaluated. Based on the results, for Class 0, the XGBoost model had the best performance, with an AUC of 84.34%. For Class 1, KNN obtained an AUC of 89.94%. For Class 2, RF model had an AUC of 71.01%. For Class 3, KNN gained an AUC of 83.08%. Finally, for Class 4, RF had an AUC of 89.10%. These findings show that the best ML algorithm in each survival class is different. The proposed RF and XGBoost models are both tree-based; therefore, they are explainable, which means they can be interpreted to make them understandable for humans, and their DTs can be easily displayed. These two attributes can help clinicians better comprehend the models’ decision-making. Furthermore, their computational cost is acceptable, and they can be easily used for large datasets.

Table 9 compares the dataset, method, outcome, performance, and explainability of two previous studies and this study. Our study and Chen’s study [14] used classification models, and Grimley et al. study [15] used a clustering model. As shown in the table, this study used a larger number of samples compared to the two other studies. Comparing the first study [14] with this study, it can be seen that the first study [14] used binary classification and predicted whether the patient would survive more than 22 months or not; however, we used multiclass classification to predict the survival intervals of patients. This range of classification provides more detailed information and planning possibilities for the clinicians. Despite using multiclass classification, the performance of our study is better than Chen’s study [14] in all criteria. Moreover, unlike Chen’s study [14], our study used an interpretation method to clarify the decision-making process of the proposed model.

**Table 9:** Comparison between previous studies and this study

Study	Dataset		Model	Class/group	Accuracy	AUC	F1-score	C-index	Explainability
Chen [14]	SEER (4,128 samples)		L2-regularized logistic regression	Binary classification (survived more than 22 months, survived less than 22 months)	0.761	0.621	0.216	-	no
Grimley et al. [15]	SEER	Dataset 1 (39,514 samples)	Ensemble Algorithm for clustering Cancer Data (EACCD)	9 epithelial ovarian carcinoma prognostic groups	-	-	-	0.7391	-
		Dataset 2 (25,291 samples, derived from dataset 1)						0.7605	
This study	SEER (42,827 samples)		Random Forest	Multiclass classification (5 classes)	0.887	0.823	0.717	-	yes

The pandemic of COVID-19 has delayed the screening, diagnosis, and treatment of cancers, including ovarian cancer, which is expected to increase their mortality rates in future [56, 57]. Nevertheless, providing a tool that can accurately predict the survival of cancer patients will enable clinicians to recognize high-risk patients, prioritize them in case of using limited resources, and make evidence-based treatment decisions for them. The ML models proposed in this study have shown satisfactory performance in predicting the survival of ovarian cancer patients. The accuracy of predicting patients’ survival and diagnosis using ML models has increased significantly since 2000 [58]. In addition, the use of interpretable ML models has been able to show better and more understandable results than statistical models [59]. ML models’ interpretability and explainability, which show the effect of each feature on the prediction and decision-making of models, increase clinical and healthcare confidence in ML models [59]. In this study, histologic type ICD-O-3 is the most important feature in the model’s decision-making and has the highest SHAP value, as it is selected as the root of the tree in three of the five cases. This feature is a code that describes the morphology and topography of the tumors [60], both of which should be considered in predicting survival and planning treatment of ovarian cancer patients, as they influence survival [61, 62]; therefore, histologic type ICD-O-3 is an effective and important feature in ovarian cancer patients’ survival prediction. The effectiveness, importance, and usefulness of this feature have also been identified in other studies [62, 63].

It is noteworthy that ML models have the ability to predict from large and complex datasets, which highlights their increasing importance [37]. The prediction provided by ML algorithms is different from the epidemiological predictions since ML algorithms predict based on individual patients’ features and not on the base of a population average. Therefore, using ML algorithms as a valuable tool in times of crisis can be very helpful to clinicians, and the results of our study contribute to realizing the availability of such a tool using ML algorithms.

### Strengths and limitations

This study has various strengths. The dataset features used were clinically meaningful and selected by our expert clinician. Correct and accurate preprocessing has been done on the dataset to avoid errors and mistakes during training and testing of ML models. For the first time, both classification and regression approaches simultaneously with multiple ML models have been implemented on the ovarian cancer patients’ dataset from the SEER database. To increase the performance of the models, the dataset has been balanced using the SMOTE method. The proposed models had an acceptable computational cost and were explainable. The SHAP method has been used to increase the confidence and clarity of clinicians in deciding on the best ML model in the classification approach. Moreover, the DTs of the best classification model of this study were drawn to provide better insight for clinicians.

However, this study has some limitations. First, it was not possible for us to implement the proposed models with more hyperparameters due to the limited resources. Second, it was not possible to validate our predictive models externally due to lack of similar available datasets.

### Implication

The main audience of this study is clinical and ML researchers who are interested in the detailed analysis of the survival of cancer patients. Since we consider explainability, our best models can be tested as a practical tool to help clinicians to get an insight about the patients’ condition. Our results have many implications for managing ovarian cancer patients. These include updating or developing clinical guidelines and protocols based on the most important factors affecting these patients’ survival. Developing a clinical decision support tool based on our results is also another possible implication. The findings of this study can also be interesting for other researchers from different fields. Furthermore, developers in the field of ML can use the findings of this study to evaluate various techniques and create prediction models.

## Conclusion

Ovarian cancer is one of the most common cancers in women. In this study, we developed ML techniques for both classification and regression approaches using the SEER dataset. An expert clinician helped us in preparing the data as well as design the classes to gain clinically meaningful results. To the best of our knowledge, our study is the first study using the regression approach for predicting ovarian cancer patients’ survival months.

In classification, RF, and in regression, XGBoost had the best performance. Both are tree-based and explainable. In addition, we considered the interpretability and transparency of the decision-making process by reporting the results using SHAP. The results of our study were promising and can be used as an auxiliary tool for clinicians to get insights into the condition of ovarian cancer patients, especially in situations like COVID-19, where a vast load goes to healthcare systems, and clinicians’ priorities would be changed.

We are currently collecting a new dataset of ovarian cancer patients in West Azerbaijan of Iran and will externally evaluate our proposed models in the future using this data.

## Data Availability

The dataset used in this study can be requested from the SEER source website at https://seerdataaccess.cancer.gov/seer-data-access.

## Appendix

See Tables 10, 11 and 12.

**Table 10:** Examined hyperparameters for proposed ML models

Model	Hyperparameters
KNN	algoritgm: (kd_tree, ball_tree, auto) p: (1, 2) n_neighbors: (1–15) other hyper-parameters values: default
SVM	kernel: (rbf, poly, linear) gamma: (0.01, 0.1, 1, 10, 50, 100) C: (0.01, 0.1, 1, 10, 50, 100) other hyper-parameters values: default
DT	spliter: (best, random) max_depth: (5, 10, 20, None) criterion: (entropy, gini) other hyper-parameters values: default
RF	criterion: (entropy, gini) max_depth: (5, 10, 15, None) n_estimators: (50, 100, 150, 200) other hyper-parameters values: default
AdaBoost	n_estimators: (50, 100, 150, 200) learning_rate: (0.5–2.0) algorithm: (SAMME, SAMME.R) other hyper-parameters values: default
XGBoost	sampling method: (gradiant_based, uniform, subsample) eta: (0.1–0.9) booster: (dart, gblinear, gbtree) other hyper-parameters values: default

**Table 11:** Performance of the RF model in all the folds of the dataset

Model	Class	Accuracy (%)	PPV (%)	NPV (%)	Sensitivity or Recall (%)	Specificity (%)	F1-Score (%)	AUC (%)
Fold 1	Class 0	88.40	68.93	93.74	75.12	91.67	71.89	83.40
	Class 1	92.10	83.96	93.90	75.27	96.36	79.38	85.82
	Class 2	81.31	53.98	87.86	51.56	88.85	52.74	70.21
	Class 3	89.69	74.19	93.45	73.25	93.74	73.72	83.49
	Class 4	91.96	77.96	95.82	83.68	94.05	80.71	88.86
Fold 2	Class 0	88.67	70.21	93.68	75.14	92.04	72.59	83.59
	Class 1	92.25	82.25	94.50	77.15	95.93	79.62	86.54
	Class 2	81.81	56.45	88.05	53.72	89.16	55.05	71.44
	Class 3	89.16	73.17	92.98	71.32	93.56	72.24	82.44
	Class 4	92.18	78.46	95.88	83.67	94.30	80.98	88.98
Fold 3	Class 0	88.49	70.53	93.36	74.22	92.12	72.33	83.17
	Class 1	92.29	83.34	94.34	77.09	96.12	80.09	86.60
	Class 2	82.08	53.42	89.20	55.14	88.52	54.27	71.83
	Class 3	89.13	74.16	92.59	69.86	93.93	71.95	81.90
	Class 4	92.32	79.73	95.74	83.57	94.56	81.60	89.06
Fold 4	Class 0	88.75	70.52	93.89	76.52	91.86	73.40	84.19
	Class 1	91.95	83.40	93.82	74.63	96.29	78.77	85.46
	Class 2	81.36	54.23	87.98	52.40	88.73	53.30	70.57
	Class 3	89.21	73.84	92.88	71.22	93.70	72.51	82.46
	Class 4	92.61	78.39	96.44	85.55	94.31	81.81	89.93
Fold 5	Class 0	88.25	68.78	93.49	74.00	91.75	71.29	82.87
	Class 1	91.87	82.88	93.86	74.94	96.11	78.71	85.53
	Class 2	81.49	52.38	88.79	53.96	88.15	53.16	71.05
	Class 3	88.89	74.87	92.17	69.15	94.00	71.89	81.57
	Class 4	91.87	77.92	95.71	83.35	94.02	80.54	88.69
*Average*	*Class 0*	*87.98*	*67.12*	*94.33*	*78.28*	*90.41*	*72.27*	*84.34*
	*Class 1*	*85.78*	*68.45*	*89.01*	*53.69*	*93.81*	*60.18*	*73.75*
	*Class 2*	*76.64*	*43.22*	*87.63*	*53.47*	*82.44*	*47.78*	*67.95*
	*Class 3*	*80.73*	*52.87*	*84.89*	*34.29*	*92.34*	*41.56*	*63.31*
	*Class 4*	*82.60*	*55.61*	*90.79*	*64.68*	*87.08*	*59.79*	*75.88*

**Table 12:** Performance of the XGBoost model in all the folds of the dataset

Fold	RMSE (%)	*R* ^2^
Fold 1	23.48	0.3061
Fold 2	19.74	0.5010
Fold 3	20.46	0.5016
Fold 4	19.32	0.5234
Fold 5	20.06	0.5016
*Average*	*20.61*	*0.4667*

See figures 7, 8, 9, 10 , 11 and 12

## Abbreviations

COVID-19: Coronavirus disease 2019
ML: Machine learning
SEER: Surveillance, Epidemiology, and End Results
RF: Random forest
XGBoost: Extreme gradient boosting
US: United States
TRIPOD: Transparent reporting of a multivariable prediction model for individual prognosis or diagnosis
SHAP: Shapley additive explanations
SMOTE: Synthetic minority oversampling technique
KNN: K-nearest neighbors
SVM: Support vector machine
DT: Decision tree
AdaBoost: Adaptive boosting
GB: Gradient boosting
PPV: Positive predictive value
NPV: Negative predictive value
AUC: Area under curve
RMSE: Root mean square error
R^2^: R-squared
